# Sale of Secret Remedies Abroad

**Published:** 1843-12

**Authors:** 


*Sale of Secret Remedies Abroad.*—An amusing scene was enacted in the Royal Court of Paris lately, during the trial before it of a Mr. Warton, the proprietor and vender of the *Ervalenta de l'Afrique* and the *Melasse de Cochinchina*, a compound respecting which, some proceedings, in an inferior court, we noticed in a former number of The Lancet. It may be recollected that on the previous occasion the ervalenta turned out to be nothing more than ground lentils, and the Cochin-chinese treacle to be ordinary molasses. The decision however, on that occasion not having been completely according to the wish of the attorney-general, he moved the case into a superior court: it will be seen that he went farther and fared worse. The following conversation before the latter court is detailed in the "Gazette de Tribunaux:"—

*Question.* You have publicly sold some alimentary substances?—*Answer.* I have sold Ervalenta and Melasse of Cochinchina.

*Q.* How have you procured the knowledge of their virtues?—*A.* It is to myself that the world owes the discovery of the quality of the first. I know nothing more efficacious against—constipation!

*Q.* But there is the melasse also.—*A.* Aye,—I should think so, and an excellent thing it is,—*pardieu!*

*Q.* Why have you not announced this remedy under its real and ordinary name?—*A.* I did so at first, but it didn't take. I then substituted the scientific for the ordinary name of it, and I have now sold all my stock.

*Q.* But you have shortened the scientific name?—*A.* I have, indeed, shortened it a little in *Frenchifying* it; but then that has nothing to do with the properties of the remedy.

*Q.* If the public knew that that which you sell is nothing but lentil-flour, probably no one would buy of you?—*A.* Well, that's possible!

*Q.* How do you sell each packet?—*A.* At three francs the kilogramme. The grocers sell it much cheaper; but I have doubled the price to avoid the appearance of any collusion with them.

*Q.* And you say that it will cure diseases?—*A.* As an article of food it is excellent. It preserves you from the *fatiguing inconvenience* of constipation. (The speaker warmed as he proceeded in detailing the virtues of his specific.) Do not believe, gentlemen, that I stand here as a fanatic—an enthusiast. No, no, I am no charlatan, and if I tell you that the Ervalenta is a specific against constipation, it is because

I have proved the fact on my own person. (General laughter.)

*Q.* What have you to say, then, of your Cochinchinese treacle?—*A.* Cochinchinese is, perhaps, not exactly the most proper word; for the article comes principally from Jamaica and Porto Rico—some little distance, indeed, from Cochinchina. I have given it that name to prevent people going to the grocers, who sell only beet-root treacle—the savages! Do you know the effect of grocers' treacle? It does not cure constipation—it increases it.

*Q.* But you make the public pay five times as dear for your melasses as they do elsewhere?—*A.* Never think it is a desire to get money that makes me sell my article so dearly. Druggists commonly get 6d for what grocers pocket only 3d. And, besides, they too often sell the vile beet-root treacle for that of the cane, that they may afterwards have to sell purgatives, &c.

Much against the wish of the attorney-general, the Royal Court acquitted Warton, who probably owed his success to his unabashed and mirth-making mode of pleading his own cause.—*Lond. Lancet,* Sept., 1843.

